# Motivations for Food Consumption during Specific Eating Occasions in Turkey

**DOI:** 10.3390/foods5020039

**Published:** 2016-05-24

**Authors:** Delores Chambers, Uyen T. X. Phan, Sirichat Chanadang, Curtis Maughan, Karolina Sanchez, Brizio Di Donfrancesco, David Gomez, Federica Higa, Han Li, Edgar Chambers, Eyyup Esen

**Affiliations:** 1 Sensory Analysis Center, Kansas State University, Manhattan, KS 66502, USA; phan.thuyxuanuyen@gmail.com (U.T.X.P.); sirichat@ksu.edu (S.C.); cmaughan@ksu.edu (C.M.); ksanchez@ksu.edu (K.S.); briziod@ksu.edu (B.D.D.); dagomezb@ksu.edu (D.G.); higa@ksu.edu (F.H.); hanl@ksu.edu (H.L.); eciv@ksu.edu (E.C.); 2 Dialog Institute of the Southwest-Kansas, 2710 S 42nd St, Kansas City, KS 66106, USA; eyyupesen@gmail.com

**Keywords:** Turkey, Turkish, motivation, food choice, dietary behavior

## Abstract

Several studies in different countries have been conducted to investigate factors affecting food choices. The objective of this study was to understand the motivations of specific food and beverage choices for different eating occasions in a typical diet of the Turkish people. A convenience sample of 141 respondents from seven different geographical regions in Turkey completed an online survey questionnaire that included questions about demographic information and details about their latest eating occasion. Respondents reported all of their motivations for choosing each food/beverage item reported for that specific eating occasion. Results indicated that different motivations played different roles in food choices of people in Turkey. Liking was a key characteristic for all eating occasions, but key natural concerns were even more important at breakfast, and need and hunger were more important for a mid-afternoon snack. Lunch involved additional motivations such as Sociability, Variety Seeking, and Social Norms. In addition to Liking, choices of different food groups were also driven by other motivations such as Habits, Convenience, Need and Hunger, Natural Concerns, and Health. This study helped better understand the current dietary patterns of Turkish people as well as the motives underlying their choices of foods and beverages for different meals and snacks. These findings could be useful for dietary campaigns that aim to improve eating behaviors in Turkey.

## 1. Introduction

Food choice is universally acknowledged as a complicated behavior that is influenced by various factors [1]. Understanding why people choose to consume certain food items in specific eating occasions is beneficial for improving people’s lives by giving dietary advice to prevent health issues such as obesity and eating disorders. Moreover, understanding motivations for eating various food categories can help facilitate new food product development and better understand marketing of these new products [2,3]. Everyday food choice depends on several factors including biology (e.g., hunger, appetite), sociology (e.g., culture, social status), physiology (e.g., mood, stress), economics (e.g., availability, budget), marketing (e.g., advertising, distribution), and consumer science (e.g., attitudes, risk perception) [1]. Among those, culture is one significant factor that shapes people’s dietary patterns and preferences [4,5]. Rules of specific cultures, subcultures, and ethnic groups are often used to create a frame of preferences, acceptances and appropriateness for foods [6]. Prescott *et al*. [7] found that health, natural content, weight control, and convenience are the most important food choice factors for the Taiwanese and (ethnically Chinese) Malaysian consumers, whereas it is price for the Japanese, and sensory appeal for the New Zealanders. Weight control, price, ethical concern, convenience, natural content, health, sensory appeal, and familiarity were found to have the same meaning and similar structural characteristics across cultures in Europe [8]. However, Pettinger *et al*. [9] reported that French respondents prioritize pleasurable and social aspects of eating and health in their food choices, while English respondents value organic issues, ethical issues, and convenience more. People in six Western Balkan countries were found to value sensory appeal, purchase convenience, health and natural content but they did not put much weight on ethical concern and familiarity [10]. In the U.S. [11] liking was clearly more important than other aspects among the general population, but other aspects, such as need and hunger, health, price, and convenience, were major secondary aspects.

Turkey is located in both Europe and Asia with the Mediterranean Sea to the south, thus it has Mediterranean nutritional influences on the coast side, and European and Asian influences in other parts of the country [12]. Previous studies have been conducted on different countries and populations regarding important factors that influence food choices, but little work has been done with the Turks so far. According to the Food and Agriculture Organization of the United Nations [13], the Turkish diet consists of three meals a day, and are based mainly on wheat products, such as breads, and also include rice, maize, legumes, and meat. Fats in the diets include olive oil in certain regions, and sunflower oil or margarine as a substitute for butter in the eastern region. Yogurt is the main dairy product consumed. Vegetables and fruits are part of the daily intake in Turkish diets, and although lamb and beef are present in Turkish cuisine, their intake has been reduced due to an increase in prices. Sweets, such as Turkish delight and baklava, also play an important role in Turkish cuisine [14]. Beside the traditional Turkish cuisine, the consumption of fast food has been increasing over the past 30 years. The younger generation of Turkish people often prefer American style fast food chains, while the older generation prefers Turkish style fast food (such as doner, kebab) [15]. Fast food consumption was also found to be related to household characteristics (size, number of children, employment of wife, socioeconomic level). Other factors such as price, health habits, and household characteristics are also influential on the type of food typically consumed. This study also found that young population in Turkey find fast food restaurants a place to socialize with friends and others.

The results from national and local surveys indicated that the average Turkish diet is sufficient to meet recommended daily intake of energy and most of the nutrients. However, consumption of animal protein, calcium, vitamin A, and riboflavin were lower than recommended [16,17]. Turkish people have been faced with two kinds of health problems: micronutrient deficiencies and diet related to chronic diseases [16]. While micronutrient deficiencies are an important problem of preschool children and child-bearing age women [16,17], obesity and coronary heart disease are more prevalent in Turkish adults [18,19]. It is important to examine the motivators of everyday food choice for Turkish people. A better understanding on their food choices is useful for developing appropriate dietary campaigns to direct them to improve their eating behavior. Knowledge of Turkish food choices can also help food manufacturers to increase a chance of success of introducing new foods into the market.

In short, this study focused on investigating the motivations of specific food and beverage choices for different eating occasions in a typical diet of Turkish people. This study was conducted as one part of a study project to understand culture, dietary behaviors, and life style of Turkish people in 2015.

## 2. Materials and Methods

### 2.1. Participants

This study was approved by the Human Subjects Institutional Review Board at Kansas State University (#7297). The participants of this study were recruited using fliers, e-mail, and word-of-mouth. E-mails were sent to more than 300 contacts throughout Turkey and flyers providing the access link to the survey were distributed by researchers to people at different locations in Turkey, such as Istanbul, Cappadocia, Ismir, Manisa, and Bursa. The Turkish people who received the e-mails and flyers were also asked to spread the word about the surveys to their extended family, friends, co-workers, employees, *etc*. To qualify for the study, the participants had to satisfy two criteria: be older than 18 years of age, and be a resident of Turkey for at least 10 years and provide consent to participate. In total, 141 respondents (out of 214 people who started the survey) completed the questionnaires. Dropout rates during the survey were higher than expected, in part because the length of the survey was long and required multiple steps.

In total, 141 respondents completed the survey, with 44% female and 56% male participants. The complete demographic breakdown can be seen in Table 1. The most common age range was 23–44, with 75% of participants falling into that category. The majority (69%) of participants were employed full-time, though 21% were unemployed, with the remaining working part-time (4%), working as a homemaker (4%), or retired (2%). Almost all of the participants (93%) were life-long residents of Turkey, with the remaining 7% having lived there for at least 10 years. This population was slightly more male, younger, about the same in income categories, and slightly less employed than the overall Turkish population.

### 2.2. Online Survey Questionnaires

**Figure 1:**
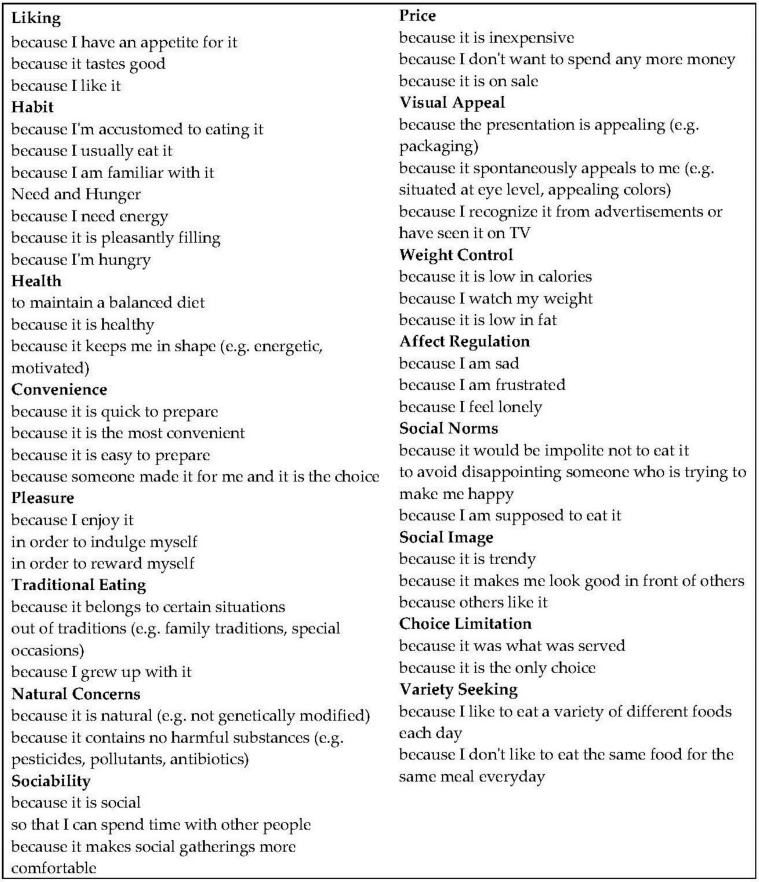
Modified version of The Eating Motivation Survey used in this study. This questionnaire included 49 motivation subscales measuring 17 motivation constructs. Reproduced with permission from Chambers and Phan, Journal of Sensory Studies, Wiley Blackwell, 2016 [11].

This study used an online questionnaire in Turkish that included questions about demographic information, such as gender, residency, occupation, and income; the latest eating occasions before completing the survey; and details about that eating occasion, such as what was eaten, where/at which time/with whom it was consumed, and how many foods and beverage items were consumed. The respondents then reported all motivations (reasons) for choosing each food or beverage item reported for that specific eating occasion using a modified version of The Eating Motivation Survey (TEMS) [3] with modification [11]. This modified version included 17 motivation constructs, *i.e.*, liking, habits, need and hunger, health, convenience, pleasure, traditional eating, natural concerns, sociability, price, visual appeal, weight control, affect regulation, social norms, social image, choice limitation, and variety seeking. Figure 1 contains the 49 subscales used to measure these 17 constructs. Another adapted modification was using a Check-All-That-Apply (CATA) procedure to collect data for motivations instead of using seven-point rating scales. This procedure was used to reduce survey burden of the respondents.

The surveys were first created in English and then translated into Turkish by a native Turkish speaker, and was double-checked by five Turkish students at Kansas State University in a discussion session to validate the translation before it was launched in Qualtrics (Qualtrics, Provo, UT, USA).

### 2.3. Data Analysis

Written results from the survey, e.g., food items listed, were translated into English by a native Turkish speaker and were categorized in food groups based on information from Turkish cooks and Internet recipes for specific dishes. Food items were recorded and classified based on the meal reported by the respondents, as well as into different food groups (e.g., dairy, grain, vegetables, fruits, or meats) using the Dietary Guidelines for Turkey [20] and the National Nutrient Database for Standard Reference Release 28 of USDA [21] for references. One difference between these two classification systems was in the dairy and eggs groups. The USDA combined dairy and eggs into one group while the Turkey dietary guidelines separated them into two groups. Since this study was investigating the dietary patterns of Turkish people, the Turkish classification was used, meaning dairy and eggs were considered as two groups for data analysis.

The 17 motivation factors from TEMS were linked to the food groups and the eating occasions using correspondence analysis (CA) [22] to extract the main motives for each eating occasion and type of food items. Hierarchical clustering also was performed on the CA factors to examine the differences/similarities in motivations among the eating occasions as well as among food groups. Proportion tests using Pearson’s chi-squared test statistic were also performed on the proportion data of all seventeen motivation constructs to validate the main motives for each food group. All analysis was performed in R 3.0.1 (R Development Core Team, Vienna, Austria, 2007) using FactoMineR package.

## 3. Results

### 3.1. Characteristics of the Main Eating throughout a Day

The most recent meal was recorded for each of the participants, with the individual food items in that meal analyzed for their motivations. For example, out of the 141 total participants, 30 (21%) reported that their most recent meal was breakfast, and so reported their motivations from those items they ate at breakfast. The number of participants reporting each meal occasion can be found in Table 1. The majority of respondents who chose breakfast as their most recent meal consumed it either before 8 a.m. (43% of the time), or between 8 and 11 a.m. (47% of the time). Lunch was generally consumed between 11 a.m. and 1 p.m. (58%) and 1 and 5 p.m. (39%). Mid-afternoon snacks were consumed between 1 and 5 p.m. (55%) and 5 and 8 p.m. (39%). Dinner was consumed between 5 and 8 p.m. by 72% of the participants, while late night snacks were consumed by the majority (67%) of people between 8 p.m. and midnight. During the period of the survey (primarily October 2015) there were no major holiday periods that would typically impact eating occasions. We did not differentiate among workdays or non-workdays in this first look at eating motivations in Turkey.

**Figure 2:**
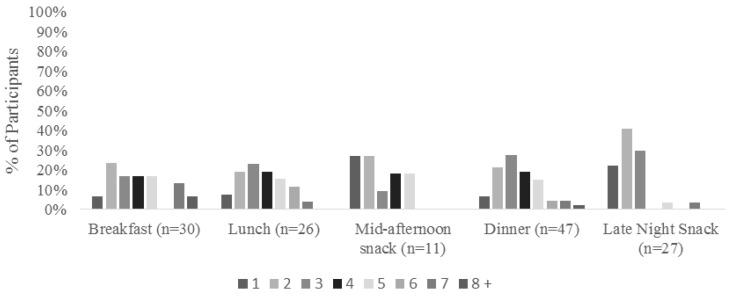
Number of items consumed during each eating occasion by participants in the study.

**Figure 3:**
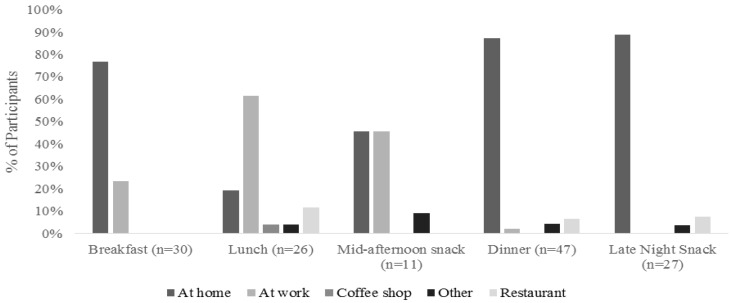
Location of eating for the participants during their reported eating occasion.

**Figure 4:**
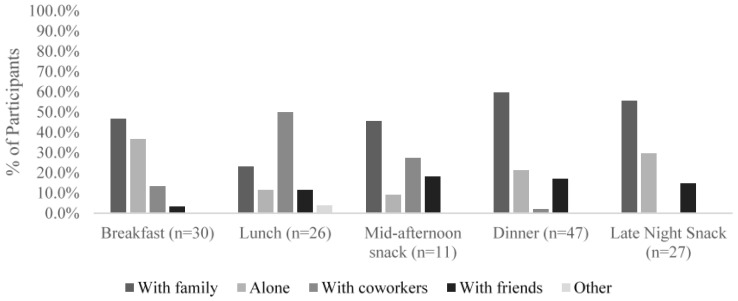
Other people present with the participants during their reported eating occasion.

Participants reported the number of food and beverage items that they consumed during their most recent eating occasion. As seen in Figure 2, there was a wide variation in the number of items consumed for each meal, though snacks tended to have fewer items in general. Participants also were asked where they consumed the previous meal, as shown in Figure 3. The vast majority of participants who said that breakfast, dinner, and late night snack was their latest meal consumed that meal at home. Lunch was more commonly eaten at work, while mid-afternoon snacks were eaten either at home or at work. Participants tended to eat breakfast, dinner, and late night snacks either with family or alone, while they typically ate lunch with coworkers (Figure 4). This follows the trend of eating location, where they are more likely to eat with family or alone when they are home, and more likely to eat with coworkers while at work.

### 3.2. Choices of Different Food Groups for Main Meals and Snacks of the Turks throughout a Day

There were 304 food items reported from 141 participants for all five eating occasions. The majority of food items (107 items) were reported for dinner followed by breakfast (77 items), late night snack (57 items), lunch (48 items), and afternoon snack (15 items), respectively. All food items were classified into 10 groups which were baked products, cereal grains and pasta, dairy, fruits, protein, soups, sweets, vegetables, tea, and water. Table 2 shows the proportion of food groups for each meal time by dividing the number of food items in each group by the total food items for each eating occasion.

Baked products, dairy, sweets, vegetables, and tea varied greatly across eating occasions with fruits and soups varying less, and items such as cereal grains, proteins, and water not varying significantly in their proportion eaten across eating occasions. Baked products, sweets, and tea were consumed mainly for breakfast and afternoon snacks. Dairy was consumed much more at breakfast than other meal times. These mostly were yogurt drinks and cheeses. Vegetables were consumed primarily for lunch, dinner, and evening snacks. Soups tended to be consumed more at dinner. Fruits seemed to be preferred for evening snacks more than the others. In addition to vegetables, lunch and dinner were associated more with water, soups, and cereal grains and pasta than other food categories. Available data about food consumption in Turkey [16,23,24] have shown that the Turkish dietary patterns are characterized by high-energy intake, with the dominance of bread and cereals, and there was a demand for meat and dairy products by Turkish households [25]. From our findings, dairy products should be increased more for lunch and dinner and protein should be the main focus for dinner. Much of this data is quite different from that found for a U.S. population [11], which revealed that coffee is the main beverage for breakfast and alcoholic drinks are only used for dinner or evening snacks while in this case, tea was the main drink for breakfast and no alcoholic beverage was reported. In addition, the U.S. people often snacked in the morning while this eating was not found for the Turkish people who participated in this study. These discrepancies showed that, as expected, eating of food groups varies depending on culture, given that 98% of Turkish population are Muslims and tea-drinking is considered a food culture in Turkey.

### 3.3. Motivations Associated with Meals and Snacks for Adult Consumers in Turkey

**Figure 5:**
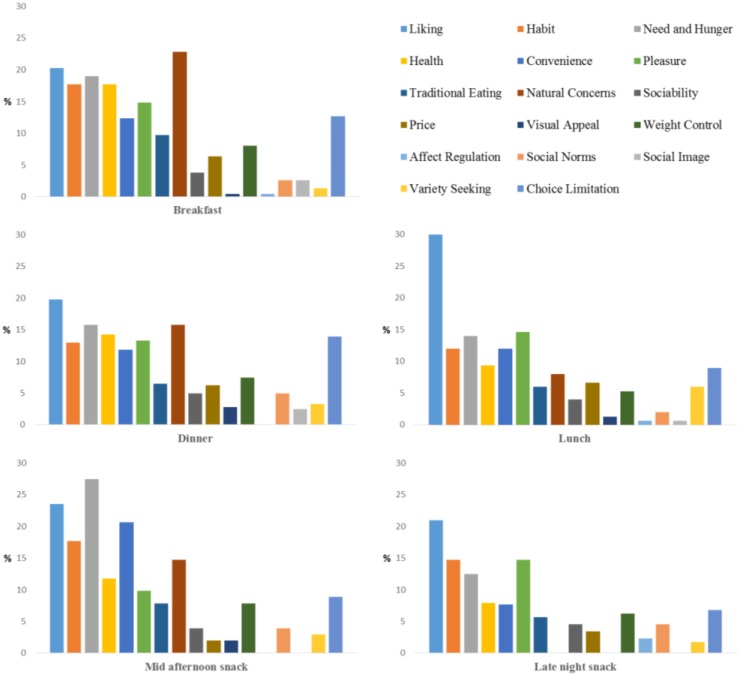
Frequency of responses (%) of 17 motivations for each eating occasion.

Figure 5 shows the frequency of responses (%) of all 17 motivations for each eating occasion. This figure also shows the main trend of motivations associated with each eating occasion. Liking (“it tastes good”, “I like it”) emerged as the main driver of food chosen for lunch, dinner, and late night snacks. It was also an important driving factor for breakfast and mid-afternoon snacks. This finding is similar to, but slightly different from findings in the U.S. using the same protocol. In the U.S. Liking was the main driver of food choice for every eating occasion with other factors different across eating occasion. In Turkey, breakfast and mid-afternoon snacks were driven by Liking, but equally or slightly more so by some other factors.

In addition to Liking, breakfast was driven by Natural Concerns (it contains no harmful substances), Need and Hunger (“I need energy”, “I am hungry”, “it is pleasantly filling”) as well as Habit and Health. Motivations for the mid-afternoon snack were driven primarily by Need and Hunger, Liking, and Convenience. The motivations for dinner, lunch and late night snacks were driven first by Liking, but then diverged somewhat with dinner choices being driven also by Natural Concerns and Need and Hunger, with lunch and late night snacks being driven by Pleasure, perhaps because of their social nature, and Need and Hunger. Motivations for snacks varied depending on the time of day with mid-afternoon snacks being driven more by convenience which late night snacks were driven more by Pleasure.

**Figure 6:**
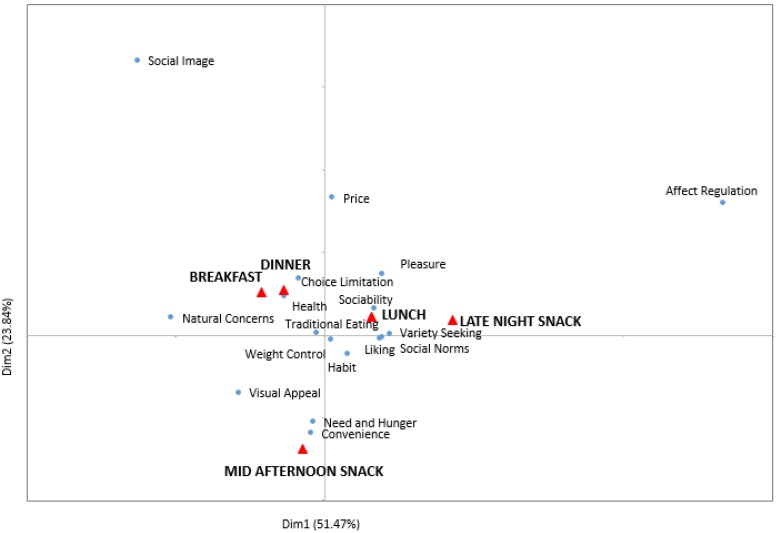
The Correspondence Analysis factor map representing five eating occasions and seventeen corresponding motivations. This factor map represents 75.31% of the total variance, with factor one contributing 51.47%, and factor two contributing 23.84% of the variance.

The CA factor map (Figure 6) shows motivations for each of the eating occasions from a multivariate perspective. This provided additional information about the motivational constructs of the five eating occasions, besides the statistically significant motivations revealed from the univariate approach above. Food choices for dinner and breakfast were also driven by Choice Limitation (“it is the only option”, “it was what was served”), Traditional Eating (“I grew up with”, “family traditions”), and Health (“to maintain a balanced diet”, “because it is healthy”, “it keeps me in shape”). The drivers for consumption of breakfast were consistent with those found by other authors [26]. Breakfast is the first meal of the day, and for this reason, people try to obtain as much energy as possible from this eating occasion. People look for filling foods that give them the energy to perform the majority of activities of the day. Most of the time breakfast is part of a routine before going to work or school, which do not give enough time for creativity. For this reason, choice limitation which was measured by “it was the only choice”, “it was what was served” was significantly reported by the Turkish respondents in this study. The dominance of dairy at breakfast and vegetables at dinner (Table 2) supported the health motivational construct of these two meals.

Food choices for lunch were additionally driven by Sociability (“I can spend time with other people”, “it makes social gatherings more comfortable”), Variety Seeking (“eat a variety of different foods each day”, “I do not like to eat the same food every day”), Social Norms (“because it would be impolite not to eat”, “to avoid disappointing someone who is trying to make me happy”, “because I am supposed to eat”), and Pleasure (“enjoy”, “reward”, “indulge myself”). This was consistent with results that reported that lunch time was related to “pleasing” and “good” [27].

The difference between the motivations for breakfast and dinner *versus* lunch could be related to the fact that Turkish people eat breakfast and dinner at home with family members and those meals are prepared by others, while lunch is consumed at work with coworkers (Figure 3 and Figure 4), which makes it a social activity. Eating at home might be the reason that choices for breakfast and dinner were influenced by Traditional Eating. Phan and Chambers [11] found Traditional Eating was only important for dinner but not breakfast from the U.S. respondents. This difference, again, showed that motivations of food choices vary depending on culture.

Convenience, Need and Hunger, and Visual Appeal (“the presentation is appealing”, e.g., packaging, “I recognized it from advertisements or have seen it on TV”) were found to be more associated with the mid-afternoon snack than the other eating occasions. According to the Foreign Agricultural Service of the USDA [28], Turkish people snack between meals. Among the products Turkish population can easily find in supermarkets or street vendors are crisps, candy bars, sweets, biscuits, Turkish delight, and crackers. Snack nuts and seeds are commonly consumed, including pistachios, peanuts, cashew nuts, sunflower seeds, pumpkin seeds, and roasted chickpeas. A popular snack among the Turkish population overall and in this study is simit, a Turkish version of a bagel that is sprinkled with sesame seeds. These foods are widely available (e.g., simit is often available on street carts in many cities), easy to buy, someone has already made it for the consumer, the snacks satisfy energy needs, and most of them are pleasantly filling.

The late night snack was close to lunch in the motivations people used to justify why they chose their specific foods. This finding was interesting given that lunch and late-night snacks did not share similar consumption patterns of where, when, what and with whom it was consumed. One possible explanation was that Turkish participants in this study did not care about Health, Weight Control, and Natural ingredients when choosing foods for these two eating occasions as much as they did for breakfast and dinner.

### 3.4. Motivations Associated with Choices of Different Food Groups for Turkish Consumers

Table 3 shows the percentages of each motivation for each food category. Liking was an important driver of consumption for all food groups. This was why the test statistic for Liking showed no significant difference among groups (*p*-value > 0.05); it was always high. People were then more likely to choose all food groups due to Habits, Need and Hunger, Health, Convenience, Pleasure, and Natural Concerns (no statistical significant difference was found between food groups). They did not significantly choose specific food items mainly because of visual appearance of the foods. Their moods and social image did not considerably affect their decision on choosing specific food items either. While Natural Concern was the main reason for choosing water, people drank tea because it was the only choice for them. People chose to eat more soups, sweets, and vegetables for the Sociability reason (social gathering, spending time with other people) than other food items. Sociability was also an important reason for the respondents to choose sweets. Results also showed that Turkish respondents consumed mainly baked products for motivations associated with mood, whether they felt sad, frustrated or lonely. Cereal grains and pasta, dairy, and also soups were consumed more than other food groups because of the Variety Seeking within these groups.

From a hierarchical clustering that was applied on the correspondence analysis factors between the food groups and the motivational constructs, six clusters of food groups were obtained according to their similarity in motivation constructs. Cluster 1 was the sweets category. Cluster 2 was water. Cluster 3 was the tea category. Cluster 4 included baked products. Cluster 5 consisted of protein, fruits and vegetable products. Cluster 6 included cereal grains and pasta, soups and dairy products. Additional information about the motivations associated with each cluster was also explored.

The sweets category (Cluster 1), which contained food items such as jam, chocolate, honey, and some sweet desserts, was predominantly associated with the Liking, and Sociability motivation constructs. Surprisingly, for Turkish participants in this study, sweets were not motivated by either Pleasure or Affect regulation (mood). Sweets are often considered “indulgent” foods, and at least for U.S. people, sweets are consumed for that reason [29]. However, in the context of this study, it did appear to be the same for the Turkish respondents. Instead, to the Turkish respondents, sweets were important for their socializing meaning with family members, given it was consumed mainly at breakfast with other family members.

Water (Cluster 2) was associated with Natural Concerns, Health, Liking, and Choice Limitation. Water intake was not associated with Visual Appearance, Social Image, Variety Seeking, Affect Regulation, and Traditional Eating. Water is a no calorie drink but of course also a bland food that has no other flavors, colors, or nutrients either. Therefore, it is no surprise to find these associations. This confirmed that the respondents were careful when answering the survey and thus provided validity for the approach used.

Consumers linked tea consumption (Cluster 3) with Liking, and Choice Limitation, whereas they showed no concern about Sociability, Visual Appearance, Weight Control, Affect Regulation or Social Image when consuming this product. Tea is a part of Turkish people’s daily life and Turkish people consume tea all day long starting from breakfast until bed time. A pot of tea will always be brewing and ready to drink in every home and workplace. Moreover, workplaces are required by law to have at least two tea breaks within the working day [30]. Therefore, the idea that tea was not associated with sociability was surprising considering the frequent drinking of tea during social situations, both in business and at home in Turkey. This may be because drinking tea during these occasions is so ingrained in the culture that consumers either did not recognize sociability as an actual reason for choosing tea or, as they suggested, it is chosen for them and is the only product available.

The baked products (Cluster 4) group, mainly breads, was linked to Liking, Need and Hunger, and Habits; and consumers did not associate baked goods with Social Norms, Variety Seeking, Visual Appearance, and Sociability motivation factors. This is the fulfilling food group in the Turkish diet. The major percentage of energy for the Turkish people comes from bread (44%) and bread with other cereals (58%) [16]. This is a nutritional problem that demands a redistribution of calorie intake to other food groups in Turkish diet. As this group of staples was associated with very “basic” motivations, any attempt to introduce new food items to substitute parts of bread consumption should deliver similar values to people. However, it would take time to move in this direction since bread is a “habit” not just a food item.

Cluster 5, including protein, fruits and vegetable products, had similar motivational patterns as seen in Table 3. Consumers associated the consumption of these food groups with Liking, Need and Hunger, Pleasure, Habits, and Natural Concerns. Motivation factors not associated with the intake of these food groups were Variety Seeking, Affect Regulation, Visual Appearance, and Social Image. Phan and Chambers [29] indicated that for a U.S. population in Kansas in the United States, choices of foods such as breakfast cereals, fruit, fruit juices, and poultry also were related to Variety Seeking. However, from this study, fruits and vegetables were more related to Habits and Needs, indicating the different role these foods have for Turkish consumers compared to U.S. consumers.

Cluster 6 included the cereal grains and pasta, soups, and dairy food groups. Motivation constructs associated with these products were Liking, Habits, Need and Hunger, Pleasure, Health, and Variety Seeking. Overall, Visual Appearance, Affect Regulation, and Social Image were not used as motivation factors by consumers. Overall, this cluster had similar motivational pattern with cluster 4 and cluster 6, except for Variety Seeking. This motivation is related to the willingness to eat a variety of foods, and in the context of Turkish eating, we learned that soups are normally consumed for dinner as a starter of the meal, and Turkish people often alter between a numbers of soups for dinner. Pilav rice is another side dish that often served to change the taste in dinner beside bread. Therefore, Variety Seeking is only applied to these specific types of foods for the Turks.

There revealed a main pattern of how Turkish respondents were motivated to choose different food groups for their diet throughout the day. For foods that are more likely to take up a big portion on the plate, such as bread, protein, grains, soups, dairy, fruits and vegetables, the motivations were related with what we called “basic needs”, which are Need and Hunger, Habits, and Health. Meanwhile, foods with smaller portion (sweets) or low calories (water, tea), the motivations are more likely associated with the other “non-basic needs”. This pattern was also observed from a population in Kansas, USA in Kansas, USA [29]. Emotions typically play a role in food choice [31]. However, for this study emotions (represented by the affect regulation construct) did not factor into the food choices of the Turkish respondents in this study.

The findings of this study were valid for the group of people with the characteristics reported in Table 1. With a convenience sample of 141 people from seven regions in Turkey, the findings of this study reflect an initial view of a much larger picture of the motivations contributing to Turkish eating. Therefore, further study with a larger population, various subpopulations, and more detail of various eating occasions is needed to verify and expand the knowledge that has been first reported in this study.

## 4. Conclusions

The study found that different motivations played different roles in food choices of the Turkish people. Breakfast and dinner shared similar patterns in food motivations which included Need and Hunger, Liking, and Natural Concerns while motivation for lunch choices involved Sociability, Variety Seeking, and Social Norms. Liking was found to be an important driving factor whether for choosing foods for meals or snacks or from a particular food group. In addition to Liking, choices of different food groups were also driven by other motivations. Habits, Convenience, Need and Hunger, Natural Concerns, and Health were equally important for all food choices, while Sociability was mainly the driving factor for soups, sweets, and vegetables. Variety Seeking was the main additional factor for grains, dairy, and soups. The Turkish participants in this study did not consider Affect Regulation or Visual Appeal as important motivations of their food choices. This study advanced the knowledge of the current dietary patterns of Turkish people, as well as the motives underlying their choices of foods and beverages for different meals and snacks. These findings could be useful for dietary campaigns that aim to improve eating behaviors of the Turks. Campaigns should be focusing on increasing the varieties of food groups in the Turks’ dietary patterns as well as promoting dairy, meat products, and non-alcoholic beverages other than tea, which may be high in sugar, for lunch and dinner. It is critical that alternative food items that promote healthful eating should deliver motivational constructs similar to those expectations of people to increase the chance of success.

## Figures and Tables

**Table 1:** Demographic information representing the participants in the study (*N* = 141).

	Demographic Information	Number of Participants	Percent of Participants
Gender	Female	69	44.0%
	Male	79	56.0%
Age	18–22	15	11%
	23–44	106	75%
	45–60	18	13%
	61–74	2	1%
Household income	<15,000 Lira	36	25.5%
	15,000–29,999 Lira	12	14.9%
	30,000–39,999 Lira	21	14.2%
	40,000–79,999 Lira	20	17.7%
	>80,000 Lira	25	8.5%
	Prefer not to answer	27	19.1%
Employment status	Employed full time	97	68.8%
	Employed part time	6	4.3%
	Homemaker	6	4.3%
	Retired	3	2.1%
	Unemployed	29	20.6%
Geographical Region	Aegean	47	33.3%
	Marmara Sea	42	29.8%
	Central Anatolia	17	12.1%
	Black Sea	15	10.6%
	Southeastern Anatolia	11	7.8%
	Mediterranean	5	3.5%
	Eastern Anatolia	4	2.8%
Most recent meal	Breakfast	30	21.3%
	Lunch	26	18.4%
	Mid-afternoon snack	11	7.8%
	Dinner	47	33.3%
	Late Night Snack	27	19.1%

**Table 2:** The proportions of different food categories consumed in each eating occasion reported by 141 Turkish respondents.

Meal	Breakfast	Lunch	Afternoon Snack	Dinner	Late Night Snack	*p*-Value *
Total Food Items	77	48	15	107	57	
**Baked products**	**18.18**	4.17	**26.67**	6.54	8.77	**0.01**
Cereal grains & pasta	2.60	12.50	6.67	12.15	12.28	0.18
**Dairy**	**22.08**	6.25	6.67	5.61	7.02	**<0.01**
Fruits	15.58	10.42	6.67	8.41	22.81	0.09
Protein	7.79	22.92	20.00	17.76	15.79	0.19
Soups	2.60	10.42	0.00	14.02	7.02	0.05
**Sweets**	**10.39**	6.25	20.00	0.93	3.51	**<0.01**
**Vegetables**	6.49	**18.75**	0.00	**24.30**	**10.53**	**<0.01**
**Tea**	**11.69**	4.17	**13.33**	0.00	7.02	**<0.01**
Water	2.60	4.17	0.00	6.54	1.75	0.46

* *p*-Value of two-sided proportion test using Pearson’s Chi-square test statistic, *df* = 5, value in bold: significant at alpha = 0.05. The numbers of food items per each group were computed into proportion by dividing by the total food items for each eating occasion.

**Table 3:** The percentages of the motivations associated with 10 different food groups reported by 141 Turkish respondents.

Number of Items (*n*) ^b^	Baked Products	Cereal Grains and Pasta	Dairy	Fruits	Protein	Soups	Sweets	Tea	Vegetables	Water	*p*-Value ^a^
	32	29	31	40	48	27	17	16	46	12	
Liking	17.71	18.39	21.51	24.17	29.86	19.75	17.65	20.83	19.57	16.67	0.42
Habits	14.58	20.69	15.05	16.67	14.58	11.11	11.76	6.25	14.49	5.56	0.41
Need and Hunger	15.63	17.24	9.68	15.00	23.61	18.52	13.73	14.58	15.22	2.78	0.10
Health	9.38	20.69	7.53	15.83	10.42	12.35	11.76	14.58	14.49	16.67	0.30
Convenience	10.94	14.66	6.45	12.50	12.50	7.41	16.18	14.06	9.24	8.33	0.37
Pleasure	9.38	14.94	13.98	15.83	17.36	9.88	13.73	6.25	12.32	13.89	0.61
Traditional Eating	8.33	4.60	3.23	9.17	10.42	6.17	7.84	8.33	5.80	0.00	0.36
Natural Concerns	12.50	13.79	8.06	11.25	12.50	11.11	17.65	3.13	17.39	25.00	0.36
Sociability	1.04	3.45	5.38	4.17	1.39	**7.41**	**15.69**	0.00	**7.25**	2.78	**<0.01**
Price	3.13	5.75	3.23	9.17	5.56	3.70	5.88	4.17	6.52	2.78	0.67
Visual Appeal	1.04	0.00	2.15	0.83	2.08	0.00	3.92	0.00	0.00	0.00	0.26
Weight Control	4.17	9.20	9.68	8.33	5.56	3.70	11.76	0.00	8.70	5.56	0.26
Affect Regulation	**4.17**	0.00	0.00	0.83	0.00	0.00	0.00	0.00	0.72	0.00	**0.01**
Social Norms	0.00	4.60	2.15	5.00	4.17	4.94	3.92	4.17	3.62	2.78	0.76
Social Image	1.04	2.30	1.08	1.67	2.08	1.23	0.00	0.00	2.17	0.00	0.94
Variety Seeking	0.00	**6.90**	**8.06**	0.00	1.04	**5.56**	0.00	3.13	1.09	0.00	**0.01**
Choice Limitation	9.38	12.07	6.45	11.25	9.38	11.11	11.76	18.75	10.87	12.50	0.92

^a^ *p*-Value of two-sided proportion test using Pearson’s Chi-square test statistic, *df* = 10, value in bold: significant at alpha = 0.05; ^b^ The sample size for Convenience was *n* × 4. Natural Concerns, Variety Seeking and Choice Limitation had sample size equal *n* × 2. The sample sizes for other motivations were *n* × 3.
